# Psychosomatic oral manifestations among pediatric patients: A cross sectional observational study

**DOI:** 10.6026/973206300221501

**Published:** 2026-03-31

**Authors:** Monika Singh, Rajendran Ganesh, Nikhil Kumar, Laxmi Bhardwaj, Aditi Singh, Pramod Machani

**Affiliations:** 1 Department of Dentistry, All India Institute of Medical Sciences, Gorakhpur, Uttar Pradesh, India; 2 Department of Preventive Dental Sciences, Faculty of Dentistry, Al-Baha University, Al-Aqiq Campus, Kingdom of Saudi Arabia; 3 Department of Dentistry, Faculty of Dental Sciences, PDM University, Bahadurgarh, Haryana, India; 4 Department of Pediatrics, Speciality Aesthetics, Paschim Vihar, New Delhi, India; 5 Department of Pediatric & Preventive Dentistry, Seema Dental College & Hospital, Rishikesh, Uttarakhand, India; 6 Department of Prosthodontics, Faculty of Dentistry, Manipal University College Melaka, Melaka, Malaysia

**Keywords:** Anxiety, bruxism, pediatric patients, psychosomatic oral conditions, stress

## Abstract

Psychosomatic oral manifestations in children are often overlooked, despite their strong association with underlying psychological stress and anxiety. This cross-sectional study evaluated 100 pediatric patients aged 6-16 years to determine the prevalence and pattern of stress-related oral conditions. Findings showed that 68% exhibited at least one psychosomatic oral manifestation, with significantly higher rates among children with moderate and high anxiety scores. Common findings included bruxism, aphthous ulcers, parafunctional habits, temporomandibular disorders and psychogenic pain, all strongly linked to elevated stress levels. This study advances existing knowledge by highlighting the need for integrated psychological screening in pediatric dentistry to improve early detection and management of psychosomatic oral conditions.

## Background:

Childhood is a dynamic developmental stage marked by rapid physical, emotional, cognitive and social changes. During this sensitive period, children are particularly vulnerable to psychological stressors arising from academic pressure, family conflicts, peer interactions, emotional trauma and environmental instability [1]. Increasing evidence shows that psychological disturbances in children can manifest as physical symptoms, a phenomenon described under psychosomatic disorders. The oral cavity, being richly innervated, highly vascular and responsive to stress-mediated hormonal pathways, frequently reflects these psychosomatic influences [2]. Psychosomatic oral manifestations are conditions where psychological or emotional factors significantly contribute to the onset, progression or exacerbation of oral signs and symptoms. While psychosomatic presentations are well- documented in adults, their prevalence and characteristics in pediatric populations remain under-recognized and under-reported, especially in developing countries [3]. Children may not possess the emotional maturity or linguistic ability to express anxiety, fear or stress verbally; instead, these emotions may surface through physical complaints, including those involving the oral mucosa, dentition, or orofacial musculature [4]. Common psychosomatic oral conditions observed in children include recurrent aphthous stomatitis, bruxism, oral habits such as lip biting and cheek chewing, geographic tongue, burning mouth sensations, temporomandibular disorders and psychogenic pain syndromes [5].

Behavioral manifestations such as nail-biting, tongue thrusting and exaggerated gag reflex may also reflect underlying emotional stress. These conditions not only affect oral health but also impair the child's quality of life by disturbing eating, speaking, sleeping and social confidence [6]. Biologically, psychosomatic conditions in the oral cavity are mediated through the hypothalamic-pituitary-adrenal (HPA) axis, autonomic nervous system and immune modulation [7]. Cortisol elevation resulting from chronic stress can alter mucosal immunity, disrupt tissue repair and enhance inflammatory responses, predisposing children to ulcerations, parafunctional habits and pain disorders. Additionally, the developing neuromuscular system and immature coping mechanisms in children heighten their susceptibility to stress-linked oral dysfunction [8]. Despite the increasing recognition of psychosomatic interactions in pediatric medicine, dentistry often lacks systematic screening protocols to identify emotional contributors to oral complaints. Many cases are wrongly treated as isolated dental problems, leading to recurrent symptoms, frustration for caregivers and unnecessary interventions. A multidisciplinary approach involving pediatric dentists, psychologists and paediatricians is essential to accurately diagnose and manage such conditions [9]. The literature reflects fragmented data regarding the prevalence, pattern and associated psychological factors of psychosomatic oral manifestations in children. Most studies focus on single conditions, such as bruxism or pathos ulcers, without comprehensively assessing multiple manifestations within a single population. Further, cultural, socioeconomic and environmental influences create variations in presentation, underscoring the need for region-specific research [10]. Therefore, it is of interest to report the prevalence, patterns and psychosocial associations of psychosomatic oral conditions among children.

## Methodology:

This cross-sectional observational study was conducted on 100 pediatric patients aged 6-16 years who reported to the Department of Pediatric Dentistry during the study period. Participants were selected using simple random sampling and only medically healthy children whose parents or guardians provided written informed consent were included. Children with systemic illnesses, neurological or developmental disorders, psychiatric conditions, those undergoing orthodontic treatment, or those taking medications for more than three months were excluded to prevent confounding influences. Demographic details including age (6-16 years), gender, socioeconomic status (low, middle, high) and academic performance (excellent, average, below average) were recorded using a structured proforma. Psychological assessment was performed using the Pediatric Anxiety Rating Scale (PARS), which ranges from 0-25; based on the total score, participants were categorized into no anxiety (0-4), mild anxiety (5-9), moderate anxiety (10-14) and high anxiety (15-25). Each child then underwent a standardized intraoral clinical examination under focused illumination to identify psychosomatic oral manifestations such as recurrent aphthous ulcers (2-10 mm in size), bruxism-related wear facets graded 0-3, lip or cheek biting lesions classified into mild, moderate, or severe, geographic tongue lesions measuring 5-20 mm, burning mouth symptoms rated on a 0-10 visual analog scale and temporomandibular joint issues assessed through mouth opening measurements ranging from 30-50 mm and palpation tenderness scored from 0-3. Parafunctional habits including nail biting, tongue thrusting, mouth breathing and psychogenic orofacial pain scored on a 0-10 pain scale were also documented. An exaggerated gag reflex was recorded and graded into mild, moderate, or severe. Psychosocial triggers were assessed through a parent-guided questionnaire covering academic stress (low, moderate, high), sleep duration categorized as adequate (8-10 hours) or inadequate (<8 hours), daily screen time classified as low (<2 hours/day), moderate (2-4 hours/day), or high (>4 hours/day) and family environment categorized as supportive, mildly stressful, or highly stressful. All findings were systematically recorded, cross-verified with parental responses and transferred to the data sheet for analysis. Statistical analysis was performed using SPSS version 25.0. Descriptive statistics were used to determine frequencies, percentages, means and ranges of psychosomatic manifestations. Associations between stress levels and oral conditions were evaluated using the chi-square test for categorical data, while continuous variables were analyzed using ANOVA. A p-value of <0.05 was set as the threshold for statistical significance. This methodology provided a uniform framework for assessing psychosomatic oral manifestations and correlating them with quantifiable psychological parameters among pediatric patients.

## Results:

A total of 100 pediatric participants aged 6-16 years were included in the study. The age group distribution showed that 47% of the children were between 6-10 years, while 53% were between 11-16 years. The sample comprised 52 males and 48 females. Psychological assessment using the PARS scale revealed that 24% of children exhibited no anxiety, 33% had mild anxiety, 28% had moderate anxiety and 15% demonstrated high anxiety levels, as summarized in Table 1 (see PDF). Overall, psychosomatic oral manifestations were observed in 68% of participants. Recurrent aphthous ulcers (2-10 mm) were present in 21% of children, while bruxism-related wear facets (Grades 0-3) were identified in 26%. Lip and cheek biting lesions were seen in 18%, geographic tongue in 10%, burning mouth sensations (VAS 1-10) in 9% and temporomandibular joint symptoms including limited mouth opening (30-40 mm) and muscle tenderness were recorded in 14%. Psychogenic orofacial pain was noted in 7% and an exaggerated gag reflex in 12% of children. These findings are detailed in Table 2 (see PDF). Parafunctional habits were highly prevalent in the study population. Nail biting was the most common, reported in 32% of participants, followed by mouth breathing (27%), tongue thrusting (19%) and parent-reported or self-reported bruxism in 22% of children. These habits are presented in Table 3 (see PDF). A strong association was identified between anxiety levels and psychosomatic oral manifestations. Only 37.5% of children in the no-anxiety group exhibited oral findings, compared to 54.5% in the mild anxiety group. This prevalence increased sharply to 85.7% in the moderate anxiety group and reached 100% in children with high anxiety levels. Statistical analysis confirmed that these differences were highly significant (p < 0.001), indicating a positive correlation between increasing anxiety levels and the presence of psychosomatic oral manifestations. These associations are shown in Table 4 (see PDF). Overall, the results demonstrate that psychosomatic oral conditions are markedly more common in children with moderate to high anxiety levels. Behavioral factors such as nail biting, mouth breathing and tongue thrusting also contributed substantially to the presentation of these conditions. The findings highlight the influence of psychological and emotional factors on oral health in pediatric patients.

## Discussion:

The present cross-sectional observational study evaluated psychosomatic oral manifestations among 100 pediatric patients aged 6-16 years and demonstrated a strong correlation between anxiety levels and the presence of psychosomatic oral conditions. A total of 68% of children exhibited at least one psychosomatic manifestation, with significantly higher prevalence among those with moderate to high anxiety scores. These findings reaffirm the close interplay between psychological stress and oral health in children. The results of this study are consistent with the observations by Mehdipour *et al.* (2023) [11], who reported a significant association between anxiety disorders and parafunctional habits such as bruxism and nail biting in children. Their research showed that children with emotional disturbances had nearly double the prevalence of bruxism compared to emotionally stable peers. This aligns with our findings wherein bruxism (26%) and nail biting (32%) were most common among the moderate and high anxiety groups. Similarly, Jürgens *et al.* (2018) [12] found that recurrent aphthous stomatitis was strongly linked to stress and emotional burden in pediatric and adolescent populations. Their study reported stress as a major precipitating factor for ulcer recurrence, supporting the present study's observation of 21% prevalence of aphthous lesions predominantly among children with elevated anxiety levels. The association between psychosocial stress and temporomandibular disorders in children has been well-documented by Alqarni *et al.* (2025) [13], who demonstrated that children experiencing academic or emotional stress exhibited higher rates of temporomandibular discomfort and limited jaw movements. The present study similarly identified temporomandibular joint issues in 14% of participants, mainly in those reporting academic pressure, inadequate sleep or family stress, suggesting that muscular tension and parafunctional habits may mediate these symptoms. Furthermore, Almutairi *et al.* (2011) [14] highlighted psychosomatic contributors to oral habits such as cheek biting, exaggerated gag reflex and mouth breathing in school-aged children. Their findings parallel the present study, where 18% of children exhibited cheek/ lip biting and 12% demonstrated exaggerated gag reflex, with both conditions showing a direct relationship with anxiety scores. Taken together, these comparative studies reinforce the evidence that stress, anxiety, emotional instability and behavioral patterns significantly influence the occurrence of psychosomatic oral manifestations in children. The findings of this study further support the need for integrated pediatric dental and psychological assessment, early identification of emotional distress and the promotion of preventive strategies addressing both mental well-being and oral health. Given that psychosomatic symptoms may serve as early indicators of psychological imbalance, pediatric dentists play a pivotal role in recognizing these signs and coordinating holistic care.

## Conclusion:

We show a strong and significant association between elevated anxiety levels and the occurrence of psychosomatic oral manifestations in pediatric patients. Children with moderate to high anxiety showed markedly higher prevalence of conditions such as pathos ulcers, bruxism, temporomandibular disorders and parafunctional habits. Early identification of psychological stressors and integrated dental-behavioral management are essential to prevent recurrence and improve overall pediatric oral health outcomes.
